# Comparison of Chronic Hemodialysis Patients under Strict Volume Control with respect to Cardiovascular Disease

**DOI:** 10.1155/2019/6430947

**Published:** 2019-07-03

**Authors:** Fadime Ersoy Dursun, Ali Ihsan Gunal, Ercan Kirciman, Ilgin Karaca, Mustafa Necati Dagli

**Affiliations:** ^1^Firat University School of Medicine, Department of Internal Medicine, Elazig, Turkey; ^2^Firat University School of Medicine, Department of Nephrology, Elazig, Turkey; ^3^Firat University School of Medicine, Department of Cardiology, Elazig, Turkey

## Abstract

**Background:**

The objective of this study was to determine the effects of strict volume control and nondipper situation on cardiovascular disease in chronic hemodialysis patients.

**Methods:**

This study is an observational and cross-sectional study including 62 patients with normotensive chronic hemodialysis using no antihypertensive drugs. A series of measurements including ambulatory blood pressure monitoring, left ventricular mass index by echocardiography, common carotid artery intima-media thickness by ultrasound, and body fluids by bioimpedance analysis were conducted for all subjects.

**Results:**

The patients were divided into two groups as dippers and nondippers according to their ambulatory blood pressure monitoring results. Average 48 h systolic, diastolic, and mean arterial blood pressure and nocturnal systolic, diastolic, and mean arterial blood pressure were significantly different between the dipper and nondipper groups (p<0.05). Before and after dialysis, extracellular fluid/intracellular fluid and extracellular fluid/dry body weight ratios were significantly higher in the nondipper group. Left ventricle mass index and interventricular septum thickness were significantly higher in the nondipper group (p<0.05). Left ventricle ejection fraction was significantly lower and common carotid artery intima-media thickness was higher in the nondipper group with a statistical significance (p<0.05). A two-predictor logistic model was fitted to the data to predict the comparability of dippers and nondippers.

**Conclusion:**

According to logistic regression analysis, the odds ratio for daytime diastolic blood pressure indicates that nondippers are 0.45 times more likely to have high blood pressure than dippers in daytime. But in night time, nondippers are about 2.55 times more likely to have high blood pressure comparing to dippers. An important finding of this study is that nondipping pattern is associated with cardiac hypertrophy and lower left ventricle ejection fraction in dialysis of patients with no hypertension. The results also suggest that applying strict volume control to achieve a normal blood pressure alone is not sufficient to reduce the risk of cardiovascular morbidity and mortality if the patients do not have a dipper status of nocturnal blood pressure.

## 1. Introduction 

Most patients receiving hemodialysis (HD) therapy due to end stage renal disease (ESRD) usually develop hypertension (HT) which is mainly systolic [1]. When HD fails to completely remove the excess fluid taken during the intradialytic period, it often causes persistent HT [2–4].

Ambulatory blood pressure monitoring (ABPM) is an automated technique that measures blood pressure at regular intervals over a period of 24 hours to 48 hours without interfering with the patient's normal daily activities. The multiple values obtained by ABPM without “alarm reactions” yield excellent measurement results [5]. Wide variations in blood pressure values in patients with HD at each session and fluctuating levels of interdialytic blood pressure indicate that interdialytic blood pressure measurements should be taken at more frequent intervals to obtain a reliable blood pressure profile. Interdialytic ABPM is the best method of assessing the blood pressure in these patients [6]. The best correlation between blood pressure and left ventricular mass index (LVMI) has been observed with ABPM. The most specific and independent determinant for LVMI and interventricular septal hypertrophy is the systolic blood pressure (SBP) load, while the best marker for the left ventricular posterior wall thickness is night ambulatory SBP [7]. Most individuals display a decreased nocturnal blood pressure of 10% or more (dippers), and those who do not display this decrease (nondippers) have an increased risk of mortality and morbidity associated with cardiovascular diseases [8].

Assessment of body composition by bioimpedance analysis (BIA) provides easy, noninvasive, safe, quick, inexpensive, and detailed information about the fluid overload in patients with HD. This technology, based on transmission of current in the human body, consists of two components: resistance induced by water and ions and reactance induced by the capacitor characteristics of the cell membranes. BIA is a useful technique for determining dry body weight in patients with HD [9, 10].

Today we know that atherosclerosis is an inflammatory disease that plays a significant role in cardiovascular disease-related morbidity and mortality in patients with chronic HD [11, 12]. The American Heart Association and the U.S. Centers for Disease Control and Prevention have published evaluation criteria for high-sensitive C-reactive protein (hsCRP) as a risk factor for cardiovascular diseases [13, 14]. High levels of homocysteine usually lead to in vitro endothelial dysfunction, oxidative damage, and thrombosis events [15]. Generally, structural and functional large artery alterations are observed in patients with ESRD. These alterations include arterial dilation, increased intima-media thickness (IMT), and greater vessel stiffness that are markers of increased risk for cardiovascular disease which is the most common cause of morbidity and mortality in patients with ESRD [16]. The objective of this study was to investigate the relationship between hypervolemia and nondipping HT in patients with chronic HD along with the association of nondipper phenomenon with cardiovascular disease markers to determine the effects of strict volume control and nondipper phenomenon on cardiovascular disease in chronic HD.

## 2. Materials and Methods

This study was conducted between May 2005 and May 2008. The study included 62 patients receiving chronic HD treatment at the HD unit of Fırat University Medical Faculty Hospital for at least 3 months. Their blood pressure levels were regulated through strict volume control, they had adopted and maintained a lifestyle with serious restriction of salt (<4-5 gr/day) to successfully allow strict volume control, and they did not receive any antihypertensive therapy. This is an observational and cross-sectional study involving HD patients, and all patients with HD were randomly selected. None of the patients had residual renal function and urine output. All patients received erythropoietin alpha treatment at a dose of 75-150 U/kg/week.

The exclusion criteria were as follows: significant heart disease, arrhythmia, cardiac pacing, presence of prosthesis, clinical manifestations of carotid artery stenosis, being hospitalized recently, and hypertension. In addition, patients with uncontrolled diabetes, obesity, sleep apnea, chronic lung disease, and pulmonary hypertension were excluded from the study. A patient was accepted to have a hypertension if his/her arterial blood pressure was measured as ≥140/90 mmHg based on at least two measurements with 5 minutes intervals while the patient was on a seated position. All the patients included in the study were weighed with the same scale before and after dialysis to calculate their intradialytic weight gain. The study protocol and the procedures were approved by Fırat University Local Ethical Committee and fully complied with the Declaration of Helsinki, and all subjects were recruited upon obtaining their informed consents.

Sympathetic activity is assessed in many studies by noninvasive techniques. Heart rate variability, BP variability, and baroreceptor sensitivity are the most frequently used methods to assess autonomic nervous system function and cardiovascular variability, a measure of the integrated control mechanisms of circulation [17]. In this study, we used noninvasive techniques such as electrocardiography, heart rate, and ABPM to evaluate the sympathetic nervous system. According to these parameters, abnormal sympathetic nervous system findings were not detected in any patient.

Patients received HD three times weekly on volumetric dialysis devices using a synthetic polysulfone membrane (Fresenius Company, Bad Homburg, Germany) and bicarbonate-based dialysate delivery system. In the standard 4-hour HD sessions three times weekly, we applied a blood flow rate of 250-350 mL/min and a dialysate flow rate of 500 mL/min. Dialysate sodium, potassium, and calcium concentrations were 133 mmol/L, 0-3.0 mmol/L, and 1.25 mmol/L, respectively. The vascular access was in the form of arteriovenous fistula in all patients. It was used with biocompatible dialyzers. All of the patients had the same temperature prescription. The ultrafiltrations of the patients were determined according to their dry weight. At the end of the dialysis session, the patients were reduced to their designated dry weight. The calculations of dialysis adequacy (Kt/Vurea) and body surface areas (BSA) were performed online by submitting the patient data, dialysis technique, and the test results on http://www.HDCN.com.

The 48-hour ABPM measurements were conducted with an oscillometric ABPM device (Suntech Medical, Morrisville, NC, USA). The monitor was applied to the patients' nonfistula arms immediately after the dialysis when the patients were in dry weight status with their heights and weights recorded. The monitors recorded readings every 30 minutes from 08:00 to 22:00 and every 60 minutes from 22.00 to 08.00 in the next morning. To obtain valid and acceptable results, we had to perform a minimum of 36-hour monitoring with readings recorded at least once an hour. Sleep-wake cycles were evaluated based on the information obtained from the patients. Intradialytic hypotension is defined as a systolic blood pressure of less than 100 mmHg or a systolic blood pressure decrease of greater than 10 mmHg or a mean arterial pressure decrease of greater than 30 mmHg with or without symptoms. Patients with intradialytic hypotension were recorded. We agreed that the finding of a nocturnal blood pressure fall of more than 10% of daytime values (night-to-day blood pressure ratio <0.9) can be accepted as an arbitrary cutoff to define patients as ‘dippers' [18].

Bioimpedance analysis is a useful technique for determining dry body weight in patients with HD. The same operator took the measurements using BIA 101-S plethysmograph (injecting 800 *μ*A and 50 kHz alternating sinusoidal current, RJL/Akern System, Florence, Italy) while BIA electrodes were placed on the body side not used for dialysis tubing in standard conditions. BIA was conducted twice a week, immediately before the last HD session of the week and 30 minutes after the session. The procedure was performed after at least 30 minutes of rest in the supine position and in a quiet environment where the temperature was between 22°C and 24°C. At each measurement, the electrodes remained attached to the device and the patient from the predialysis period until the end of the postdialysis period. Based on a fluid model using these resistances, extracellular fluid (ECF), intracellular fluid (ICF), and total-body fluid (TBF) are calculated. These volumes then are used to determine the amount of fluid state. All calculations are performed automatically by the software of the Body Composition Monitor [19].

The same cardiologist conducted the carotid Doppler and echocardiographic examination of the patients at the beginning of each HD session in the midday of the short interdialytic days. The teleradiographic images were taken before dialysis on the day of the examination. Arterial compliance was calculated by the formula (AC) = Π[D (s)^2^ –D (d)^2^]/{4[P(s)—P(d)]}/ 7,6 (mm^2^/kPa). [D (s): carotid systolic diameter (mm), D (d): carotid diastolic diameter (mm), P (s): SBP (mmHg), P(d): diastolic blood pressure (DBP) (mmHg)].

The predialysis and postdialysis blood samples were taken after the short interdialytic day. The samples were analyzed on the same day by our hospital's central laboratory without delay. During the study, we conducted routine hematology, biochemistry, coagulation, and immunology tests, as well as parathyroid hormone and hs-CRP tests.

The research data obtained from the groups were analyzed using SPSS 21.0 for Windows (SPSS Inc., Chicago, IL, USA). The data were checked with the Shapiro-Wilk test, where normal distribution was not observed, and it was accepted that if p>0.05 the data were not normal, while if p<0.05 the data were considered normal. The data obtained during the study were presented as mean ± standard deviation. Simple* t*-test was used to compare the data between groups. The correlation between some parameters in patients was analyzed by Pearson's correlation test. To explain which independent variable (among the given variables) correctly predicts the dependent variables of dippers and nondippers, the logistic regression was employed. Logistic regression is used to predict the outcome based on values of a set of predictor variables. It is similar to a linear regression model but is suited to models where the dependent variable is dichotomous. Logistic regression coefficients can be used to estimate odds ratios for each of the independent variables in the model (SPSS Inc., Chicago, IL, USA). For all tests, a* p* value of ≤ 0.05 was considered statistically significant.

## 3. Results

A total of 62 patients were included in this study. Of these 62 patients, 19 had secondary chronic pyelonephritis/urinary system infection, 18 had glomerulopathy, 15 had secondary kidney stone, and the remaining 10 had other diseases. Sleep apnea and pulmonary hypertension were not present in any of our patients. Two years after completion of our study, none of our patients had died from cardiovascular events, and only 1 out of 62 patients died from infection. The patients were divided into two groups as dippers and nondippers according to their ABPM results. Basic patient demographic characteristics and laboratory data are shown in Table 1. Hemoglobin, calcium, and albumin values were higher with a statistically significant level in the dipper group than those of the nondipper group. However, hsCRP and phosphorus levels were significantly higher in the nondipper group comparing to the dipper group. No statistically significant difference was found between nondippers and dippers in other parameters. There was no evidence of sympathetic nervous system activity disorder in clinical, electrocardiography test and ABPM results in all patients.

Ambulatory blood pressure monitoring results of the dipper and nondipper patients are shown in Table 2. Initial manual SBP, DBP, average 48 h SBP, average 48 h DBP, 48 h mean arterial pressure (MAP), nocturnal SBP, nocturnal DBP, and nocturnal MAP were statistically different between the dipper and nondipper groups. But there was no statistical difference between the dipper and nondipper groups in other parameters. None of our patients developed intradialytic hypotension. Systolic dipping (%) was 2.75 ± 5.70 in the nondipper group and 13.56 ± 5.99 in the dipper group which was a significant difference as expected. Similarly, diastolic dipping was 4.29 ± 2.74 in the nondipper group and 14.69 ± 5.11 in the dipper group, and a statistically significant difference was detected between them. The systolic and diastolic differences clearly emphasized the significance of blood pressure between these groups.

The results of BIA are shown in Table 3. Predialysis and postdialysis ECF/ICF ratios and postdialysis ECF/dry body weight ratios were significantly higher in the nondipper group, suggesting an association between ECF and nondipper phenomenon. However, there was no statistically significant difference between other parameters.

The results of echocardiography and carotid artery Doppler examination are shown in Table 4. LVMI was 115.04 ± 30.10 in nondippers and 98.12 ± 22.25 in dippers. LVMI was significantly higher in the nondipper group (p=0.026). Left ventricle ejection fraction (LVEF) was 57.11%  ± 8.00 in nondippers and 64.29%  ± 7.30 in dippers. LVEF was significantly lower in the nondipper group (p=0.001). Interventricular septum (IVS) thickness was 1.25 ± 0.27 in the nondippers and 1.03 ± 0.16 in dippers. IVS was significantly higher in the nondipper group (p=0.001). Common carotid artery IMT was 0.18 ± 0.01 in the nondipper group and 0.11 ± 0.01 in dipper group. These values were higher in the nondipper group with a statistical significance. Arterial compliance values of the nondipper and dipper groups did not show a statistically significant difference.

**Figure 1:**
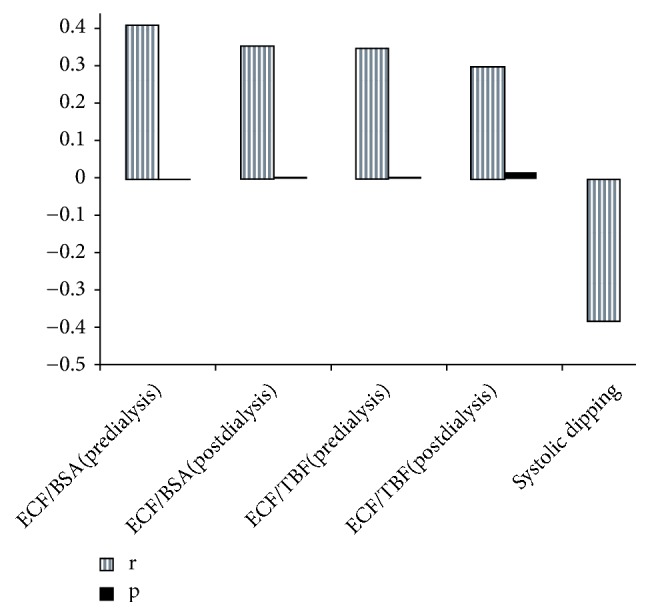
The correlation between LVMI and bioimpedance parameters and systolic dipping.

The correlation between some important parameters is shown in Table 5 and Figure 1. The Pearson's correlation test was conducted to determine the parameters associated with systolic dipping in all patients and revealed that the decline rate in SBP was negatively correlated with carotid IMT, LVMI, ECF/BSA (postdialysis), ECF/TBF (pre- and postdialysis), and hsCRP. A two-predictor logistic model was fitted to the data to predict the comparability of dippers and nondippers among other variables. The predictor variables were daytime and nighttime diastolic blood pressure. A test of the model was statistically significant (*χ*^2^  _(2)_ =55.15; p<0.05).  Table 6 shows the logistic regression coefficient, Wald test, and odds ratio for each of the predictors. The odds ratio for daytime diastolic blood pressure suggested that when holding all other variables constant, a nondipper is 0.45 times more likely to have high blood pressure comparing to a dipper in daytime, while in the night time, a nondipper is about 2.55 times more likely to display high blood pressure than that of a dipper. Both results were found statistically significant.

## 4. Discussion

The most important finding of this study is that nondipping pattern is associated with cardiac hypertrophy and lower LVEF in dialysis of patients with no HT. The findings also suggest that a salt-restricted diet and a strict volume control are very effective to control the blood pressure of chronic HD patients. According to our results, a normal blood pressure (normotensive) of a patient is not enough to reduce the risks of cardiovascular diseases, but a dipper nocturnal blood pressure profile is also needed. The present study was set up to use an established method for determining body composition, hydration, and blood pressure status in chronic HD patients. Demographic data of patients and some laboratory findings are shown in Table 1. Hemoglobin, calcium, and albumin values were significantly higher in the dipper group than in the nondipper group. However, hsCRP and phosphorus levels were higher in the nondipper group. As commonly known, low levels of calcium are associated with poor renal outcome in chronic kidney disease (CKD) [20], and high levels of phosphorus are associated with mortality in CKD [21]. Volumetric control in nondipper patients is worse according to BIA results (Table 3), suggesting that tight volume control may regulate disease and thus prognosis may well affect the direction. Another major underlying factor that plays a role in atherogenesis and pathogenesis of major cardiovascular complications in the general population is chronic inflammation [22]. High hsCRP is indicative of inflammation, suggesting that the inflammatory process continues in nondipper patients. Therefore, nondipper patients may be more likely to have a cardiovascular disease risk.

While the centers using antihypertensive medication for HT during the treatment of chronic renal failure have reported failure in 75% of the patients despite using triple or quadruple drug combinations, the centers implementing the method of strict salt and volume control have reported achieving and maintaining normal blood pressure in over 90% of the patients [3, 4]. It is well established that changes occur in circadian rhythm of blood pressure and HT in patients with chronic renal failure [23]. Patients with nocturnal decline in either SBP or in DBP of 10% or more are classified as dippers, while those with nocturnal reduction of less than 10% are classified as nondippers [24]. The ABPM measurements have shown that blood pressure levels in patients with HD do not decline at night and that there is a higher “nondipping” prevalence among them [25]. As shown in Table 2, when the 48-hour ABPM results were analyzed, the beginning manual SBP, DBP, mean 48 hours' SBP, DBP, MAP, night SBP, DBP, and MAP values were significantly lower in the dipper group than those of the nondipper group. There was no evidence of sympathetic nervous system activity disorder in clinical, laboratory, and ABPM results. Although there were no significant HT values in both groups, lower blood pressure parameters in the dipper group suggested that tight volume control positively affects blood pressure values.

Numerous studies on chronic renal patients have examined the association between HT and fluid/salt loading by BIA method [26]. In the current study as well, BIA was performed in the patients with HD. In our study, the volume levels of the patients were measured by BIA. Table 3 shows the BIA results of both groups. The higher rates of predialysis and postdialysis ECF/ICF ratios and postdialysis ECF/dry weight ratio in the nondipper group indicate that extracellular excess volume is present in the nondipper group. This excess volume may also be considered responsible for the nondipper event.

M-mode echocardiography is a precise diagnostic tool for predicting left ventricular hypertrophy, dilatation, and systolic and diastolic dysfunction [27]. LVMI, left ventricular volume index, and left ventricular volume fluctuations can be calculated in patients undergoing dialysis [28]. Some publications have reported that ABPM provides the best prediction regarding the correlation between blood pressure and LVMI [7]. The relevant research in the literature has reported that the nondipping status in patients with essential HT is associated with left ventricular hypertrophy, stroke, and cardiovascular morbidity and mortality [24]. For this purpose, we measured the LVMI, IVS, and LVEF of the patients by echocardiography and detected higher LVMI and IVS values in the nondippers than those of the dippers. On the other hand, LVEF values were higher in dipper patients comparing to the nondipper ones. Therefore, it is suggested that the LVMI and IVS increase while LVEF decreases in patients with HD, especially in the nondipper patients. It is a noteworthy finding that although all patients included in the study had normal blood pressure and did not use antihypertensive drugs, there was an increase in their LVMI and IVS values and a decrease in their LVEF values, which occurred mainly in the nondipper group.

Ultrasound measurements of common carotid artery (CCA) IMT are considered a reliable approach that can be used in the studies aiming to identify cardiovascular risk factors [29]. London et al. were the first to show that CCA IMT was associated with posterior wall thickness and LVMI [27]. In our study, we also measured CCA IMT and arterial compliance using Doppler ultrasonography to identify the cardiovascular risk status in patients with HD and to detect the association of CCA IMT with hypervolemia and inflammation. Consistent with the results reported in the literature, we found that CCA IMT values were significantly increased in the patients. The lower ratio of ECF/ICF in the dipper group suggests that the finer volume control of the carotid IMT is more effective in this group.

We also examined the correlation between some parameters in all patients, including both patient groups (Table 5). Negative correlations were found between systolic dipping and carotid IMT thickness, LVMI, ECF/BSA, ECF/TBF, and hs-CRP. This means that IMT and LVMI will be thinner and ECF and hs-CRP will be lower in dipper patients. A two-predictor logistic model suggested the comparability of dippers and nondippers among other variables and confirmed significant difference of nondippers' higher blood pressure both during daytime (0.45 times more likely to have high blood pressure comparing to dippers) and during the night time (2.55 times more likely) (Table 6).

In conclusion, we emphasize in this study that the tight volumetric control is effective. The findings of this study suggest that nondipping pattern may generate cardiac hypertrophy and a lower LVEF in dialysis of patients even if they do not have HT. The findings of the current study are in accordance with earlier observations in the literature suggesting that a salt-restricted diet not restricting water intake and strict volume control are essential for controlling the blood pressure in patients with chronic HD. However, we also observed that having normal blood pressure through strict volume control is not sufficient to reduce cardiovascular mortality and morbidity in these patients; they should also have a dipper nocturnal blood pressure profile in addition to being normotensive. Hence, our findings clearly suggest that HD patients should be strictly checked for this hidden blood pressure to avoid cardiovascular mortality and morbidity risks.

Although we had some limitations including medications, genetic background, and nutrition interaction of the patients, the results of the current study have also produced other important questions that remain unanswered to date: Do the elevated hsCRP levels in nondipper patients with HD result from the excess ECF volume? Is the microinflammation detected by hsCRP measurements an underlying cause of nondipper status? Is it reasonable to suggest that chronic microinflammation can be prevented by implementing strict volume control and that patients with ESRD can avoid cardiovascular events by applying this method? Further studies are required to address these questions.

## Figures and Tables

**Table 1:** Basic patient demographic characteristics and laboratory data.

Parameters		Nondipper (n=38)	Dipper (n=24)	p
Gender	Male n (%)	21 (55.3)	12 (50)	ns
	Female n (%)	17 (44.7)	12 (50)	ns

Diabetes mellitus	Yes n (%)	9 (23.7)	7 (29.2)	ns
	No n (%)	29 (76.3)	17 (70.8)	ns

Parameters		(Mean ± SD)	(Mean ± SD)	P

Age (year)		54.95 ± 15.62	54.17 ± 9.67	0.826

Height (cm)		159.74 ± 8.55	162.88 ± 11.10	0.366

Weight (predialysis, kg)		57.66 ± 12.42	61.74 ± 12.34	0.109

Dry weight (postdialysis, kg)		56.64 ± 12.08	60.43 ± 12.06	0.235

BSA (m^2^)		1.57 ± 0.19	1.63 ± 0.21	0.101

Duration of hemodialysis (months)		51.13 ± 51.22	54.75 ± 39.08	0.769

Kt/Vurea		1.47 ± 0.37	1.41 ± 0.59	0.107

nPCR (gr/kg/day)		1.26 ± 0.38	1.22 ± 0.37	0.704

Hemoglobin (gr/dL)		10.84 ± 1.42	11.29 ± 1.18	0.201

PTH (pg/mL)		284.05 ± 244.78	315.04 ± 242.00	0.628

hsCRP (mg/L)		3.93 ± 3.67	1.71 ± 1.21	0.006

Ca (mg/dL)		9,12 ± 0.85	9,69 ± 0,81	0.012

P (mg/dL)		6,75 ± 0.92	6,09 ± 0.46	0.002

Albumin (gr/dL)		3.91 ± 0.42	4.13 ± 0.42	0.03

Homocysteine (umol/L)		26.89 ± 15.57	20.22 ± 5.60.	0.049

SD: standard deviation, BSA: body surface area, nPCR: normalized protein catabolic rate, PTH: parathormone, hsCRP: high sensitivity C-reactive protein, Ca: calcium, and P: phosphorus.

**Table 2:** Results of ambulatory blood pressure monitoring in nondipper and dipper patients.

Parameters	Nondipper (n=38)	Dipper (n=24)	p
	(Mean ± SD)	(Mean ± SD)	
Beginning manual SBP (mmHg)	118.74 ± 15.12	105.42 ± 21.46	0.006

Beginning manual DBP (mmHg)	74.61 ± 15.10	65.83 ± 17.17	0.027

After dialysis manual SBP (mmHg)	97.37 ± 21.27	95.42 ± 21.87	0.654

After dialysis manual DBP (mmHg)	61.32 ± 15.10	58.75 ± 14,54	0.295

Average 48 h SBP (mmHg)	121.86 ± 12.83	112.92 ± 13.90	0.012

Average 48 h DBP (mmHg)	75.88 ± 10.82	64.58 ± 8.41	0.0001

Average 48 h MAP (mmHg)	91.24 ± 10.66	81.89 ± 11.33	0.002

Daytime SBP (mmHg)	122.36 ± 13.54	120.30 ± 18.57	0.615

Daytime DBP (mmHg)	76.51 ± 11.10	74.63 ± 16.31	0.589

Daytime MAP (mmHg)	91.75 ± 10.73	90.00 ± 16.46	0.613

Nocturnal SBP (mmHg)	119.18 ± 11.89	106.73 ± 16.27	0.001

Nocturnal DBP (mmHg)	74.20 ± 10.84	63.42 ± 13.39	0.001

Nocturnal MAP (mmHg)	89.38 ± 10.13	78.19 ± 13.81	0.001

Systolic dipping (%)	2.75 ± 5.70	13.56 ± 5.99	0.0001

Diastolic dipping (%)	4.29 ± 2.74	14.69 ± 5.10	0.0001

Average 48 h HR (beat/min)	77.20 ± 8.82	76.31 ± 8.15	0.693

Average daytime HR (beat/min)	79.92 ± 9.02	80.02 ± 9.46	0.967

Average nocturnal HR (beat/min)	72.49 ± 8.47	68.19 ± 6.75	0.040

SD: standard deviation, SBP: systolic blood pressure, DBP: diastolic blood pressure, MAP: mean arterial pressure, and HR: heart rate.

**Table 3:** Results of bioimpedance in nondipper and dipper patients.

Parameters	Nondipper (n=38)	Dipper (n=24)	p
	(Mean ± SD)	(Mean ± SD)	
Predialysis TBF (lt)	30.80 ± 6.9	33.00 ± 6.3	0.385

Postdialysis TBF (lt)	29.2 ± 6.56	31.00 ± 6.04	0.382

ECF/ICF (predialysis)	1.08 ± 0.33	0.95 ± 0.30	*0.045*

ECF/ICF (postdialysis)	0.98 ± 0.30	0.84 ± 0.26	*0.025*

ECF/BSA (predialysis)	9.90 ± 1.70	9.54 ± 1.17	0.829

ECF/BSA (postdialysis)	8.9 ± 1.8	8.43 ± 1.16	0.205

ECF/TBF (predialysis)	0.51 ± 0.1	0.48 ± 0.07	0.227

ECF/TBF (postdialysis)	0.46 ± 0.8	0.42 ± 0.07	0.110

TBF/BSA (predialysis)	19.49 ± 2.32	20.11 ± 2.10	0.472

TBF/BSA (postdialysis)	18.43 ± 2.23	18.92 ± 1.95	0.368

TBF/dry weight (predialysis)	0.55 ± 0.06	0.56 ± 0.08	0.787

TBF/dry weight (postdialysis)	0.52 ± 0.06	0.52 ± 0.07	0.953

ECF /dry weight (predialysis)	0.28 ± 0.04	0.26 ± 0.03	0.086

ECF /dry weight (postdialysis)	0.25 ± 0.04	0.23 ± 0.03	*0.046*

SD: standard deviation, TBF: total body fluid, BSA: body surface area, ECF: extracellular fluid, and ICF: intracellular fluid.

**Table 4:** Results of echocardiography and carotid Doppler ultrasound in patients.

Parameters	Nondipper (n=38)	Dipper (n=24)	p
	(Mean ± SD)	(Mean ± SD)	
LVMI (gr/m^2^)	115.04 ± 30.10	98.12 ± 22.25	*0.026*

LVEF (%)	57.11 ± 8.00	64.29 ± 7.30	*0.001*

IVS thickness (mm)	1.25 ± 0.27	1.03 ± 0.16	*0.001*

Carotid IMT (mm)	0.18 ± 0.01	0.11 ± 0.01	*0.025*

Carotid AC (mm^2^/kPa)	1.12 ± 0.58	1.28 ± 0.68	ns

SD: standard deviation, LVMI: left ventricle mass index, LVEF: left ventricle ejection fraction, IVS: interventricular septum, IMT: intima-media thickness, and AC: arterial compliance.

**Table 5:** The correlation between some parameters in all patients.

Parameters		r	p
Systolic dipping	Carotid IMT (mm)	-0.260	*0.041*
	LVMI (g/m^2^)	-0.380	*0.002*
	ECF/BSA (postdialysis)	-0.251	*0.049*
	ECF/TBF (predialysis)	-0.328	*0.009*
	ECF/TBF (postdialysis)	-0.342	*0.007*
	hsCRP (mg/L)	-0.282	*0.026*

IMT: Intima-media thickness, LVMI: left ventricle mass index, ECF: extracellular fluid, BSA: body surface area, TBF: total body fluid, and hsCRP: high sensitive C-reactive protein.

**Table 6:** Logistic regression analysis predicting dippers and nondippers from daytime and nighttime blood pressure.

predictors		B	S.E.	Wald (*χ*^2^)	df	Sig.	exp(B)	95% C.I. for exp(B)	
								Lower	Upper
Step 1^a^	daydbp*∗*	-0.795	0.237	11.281	1	0.001	0.452	0.284	0.718
	nightdbp*∗∗*	0.935	0.271	11.903	1	0.001	2.548	1.498	4.334
	Constant	-4.248	2.877	2.180	1	0.140	0.014		

a. Variable(s) entered on step 1: daydbp; nightdbp; Cox&Snell R^2^: 0.59; Nagelkerke R^2^: 0.80; -2Log likelihood: 27.61.

*∗*Day time diastolic blood pressure. *∗∗*Night time diastolic blood pressure.

## Data Availability

The data used to support the findings of this study are available from the corresponding author upon request.
